# Cases in Practice

**Published:** 1868-03-01

**Authors:** W. Anderson

**Affiliations:** Leroy, Ill.


### CASES IN PRACTICE.

BY W. ANDERSON, M.D., LEROY, ILL.

### CASE I.

*Report of a Case of Instrumental Delivery.*—January 23, 1868, called, in consultation with Dr. D., to attend a lady, *æt.* 36, who had given birth to seven children. Dr. D. informed me that when he was called in, labor had progressed for twelve hours; the membranes being ruptured, and the amniotic fluid having escaped. It was a shoulder presentation, with the head in the left acetabulum, the left arm protruding from the vagina, and very much swollen, so that the insertion of the hand into the uterus was impossible, while the force of the uterus was so great as to prevent an adjustment of the fœtus by bringing the head into the pelvis. The arm could not be replaced, and we decided an amputation unavoidable; after performing which, at the shoulder, the feet were brought down with some difficulty, thus saving any further mutilation of the infant, which, however, we knew to be dead before the operation. The knowledge of its death had no influence on our action, and had it been alive, it would have been born alive, in all probability, with one arm. Can some sage practitioner suggest a method of procedure by which such a danger may be avoided?

CASE II.

The well known pestiferous affection, so common in the West, and universal in the army, variously known as prairie itch, ground itch, army itch, etc., may easily be cured by such irritants or caustics as diluted sulphuric acid, corrosive sublimate, or white vitriol; or, what is more certain, the three combined, in the form of ointment, or solution; while all such applications as blue ointment, or Fowler's solution, are useless, or nearly so. This fact may be of use to many physicians, or all who use the latter remedies. The disease, which is exceedingly annoying, can be cured only by being killed in this way, and can not be neutralized, or driven from the system, or cured by alteratives, as many suppose. It is only second cousin to scabies, and *Sulphur* has no effect on it.

